# Green tea catechins in combination with irinotecan attenuates tumorigenesis and treatment-associated toxicity in an inflammation-associated colon cancer mice model

**DOI:** 10.1186/s43046-021-00074-4

**Published:** 2021-07-26

**Authors:** Gaurab Borah, Manuj Kumar Bharali

**Affiliations:** Cell & Molecular Biology Section, Department of Zoology, Rajiv Gandhi University, Rono Hills, Doimukh, Itanagar, Arunachal Pradesh 791112 India

**Keywords:** Green tea, Irinotecan, Colon cancer, Anticancer, Toxicity

## Abstract

**Background:**

Administration of green tea (GT) catechins has been reported to ensue antitumor activity in combination with chemotherapeutic drugs against different cancer types. Irinotecan (IRN) is a highly effective chemotherapeutic drug against various types of cancer including colon cancer along with its analogous dose-limiting side effects viz. diarrhea, neutropenia, leucopenia, and non-alcoholic fatty liver disease (NAFLD) as major toxicities.

**Methods:**

In this study, we investigated the antitumor effects of GT alone or in combination with IRN in inflammation-associated colon cancer mouse model induced by azoxymethane (AOM) and dextran sulfate sodium (DSS). We also evaluated the effect of GT- on IRN-induced toxicity and histopathological alterations. Animals were divided into six groups (*n* = 5 per group). After induction of cancer model, animals were treated with GT and/or IRN. We observed the inflammation, tumor progression, and ameliorative effects of GT and IRN alone or in combination.

**Results:**

Because of antioxidant potential of GT, IRN-induced toxicity ameliorative effect of GT was also studied in combined treated groups. It was found that co-administration of IRN and GT significantly decreased number of tumors and simultaneously was found to ameliorate diarrhea along with leucopenia and neutropenia. Besides these, mitigation of adenomatous characters and NAFLD was also observed in the IRN- and GT-treated group when analyzed histologically.

**Conclusions:**

GT significantly reduced the toxicity induced by IRN in terms of diarrhea, neutropenia, leucopenia, and NAFLD and works as an effective anticancer agent as it mitigates histopathology of colon adenocarcinoma.

**Graphical abstract:**

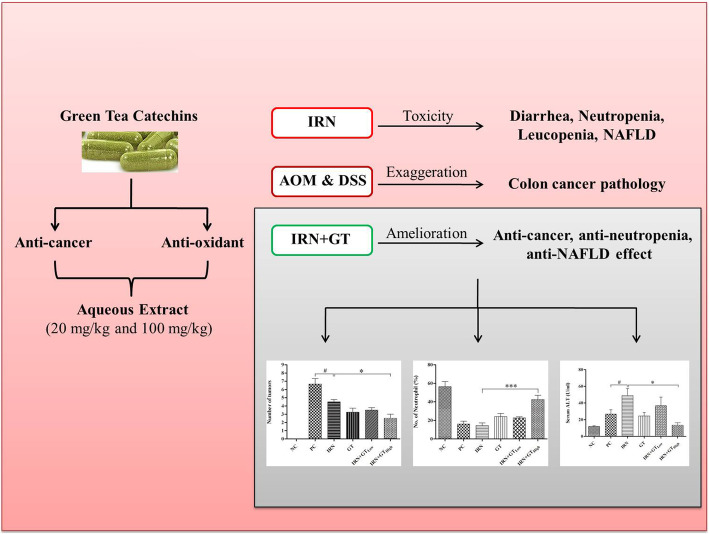

## Background

Cancer is a globally distributed leading fatal disease characterized by unconfined development and proliferation of cells causing millions of demise in recent years [1]. According to the World Health Organization (WHO), an estimate of about 15 million contemporary cancer incidents was being predicted in 2020 [2]. Among these, colorectal cancer accounts the third major prevalence with an approximate annual mortality rate of 9.4% [3]. In an Indian scenario, colon cancer ranks 8th, but reported cases were diagnosed mostly in young people, higher proportions of signet ring carcinomas and patients representing advanced stage which may be due to inadequate access to healthcare and socioeconomic factors [4]. Irinotecan (IRN), a chemotherapeutic agent, is reported to be effective in manifestation of metastatic colorectal cancers together with other cancer types [5]. However, treatment with IRN is accompanied by certain dose-limiting toxicities inducing varied toxic events including diarrhea, leucopenia, and neutropenia [5, 6]. Elevation in the level of serum transaminases, alkaline phosphatase, bilirubin, creatinine, and treatment-associated non-alcoholic fatty liver disease (NAFLD) are other common side effects of IRN [6, 7].

Nutritional supplements are now commonly integrated into therapeutic strategies. This approach can overcome the disadvantages of monotherapy and enhance therapeutic effects [8]. Dietary amalgamations ameliorate the activities of cytotoxic agents and alleviate its antagonistic effects [9]. Therefore, introduction of nutritional approach in mitigation of the detrimental counter-effects induced by IRN eliminating dose-limiting intestinal toxicity has been a prime interest. Green tea (GT) is a highly consumed beverage processed from the leaves of *Camellia sinensis*, which is extensively studied for its anticancer effects [10] and drug-induced toxicity ameliorative effects [8, 11]. The major polyphenols in GT, generally known as catechins, are (-)-epigallocatechin-3-gallate (EGCG), (-)-epigallocatechin (EGC), (-)-epicatechin-3-gallate (ECG), and (-)-epicatechin (EC) [10]. In animals, polyphenols of GT extracts promotes inhibitory effects against the development of tumor formation at different organ sites viz. skin, oral cavity, esophagus, mammary gland, lung, liver, pancreas, stomach, intestine, colon, bladder, and prostate cancers. In addition to suppressing cell proliferation, promoting apoptosis, and modulating signaling transduction, GT polyphenols, especially (-)-epigallocatechin-3-gallate, also inhibit cell invasion, angiogenesis, and metastasis [10]. This approach can overcome the disadvantages of monotherapy and enhance therapeutic effects [8]. Polyphenols of GT vary in structure and function but can enhance the effect of drugs synergistically up to 10–15 times by reducing its toxicity. GT catechins with anticancer agents are more effective than monotherapy [11]. Combining with chemotherapeutic drugs GT catechins may decrease chemotherapy associated side effects of IRN and increase efficacy of the treatment due to chemopreventive potential. Hence, this study is designed to study the efficacy and toxicity amelioration potential of GT in combination with IRN in colon cancer mouse model.

## Methods

### Chemicals

Azoxymethane (CAS number: 25843-45-2) and dextran sulfate sodium salt (CAS number: 9011-18-1) were purchased from Sigma-Aldrich, USA. IRN (Imtus) (CAS number: 136572-09-3) was purchased from Emcure Pharmaceuticals Limited, India, and other test chemicals and stains were purchased from Merck, India. The spectrophotometric kit for alanine transaminase (ALT) and creatinine estimation were purchased from Coral, India. The rest of the chemicals used in the study were of analytical grade purity and obtained locally. GT 400 mg capsules were purchased from Zenith Nutrition India limited and the extract was prepared in distilled water for the treatment.

### Extract preparation

GT capsules were used for the treatment along with IRN administration and it was used directly as has been purchased commercially. Four hundred milligrams of GT content was encapsulated in the capsule supplemented with 65% polyphenols, 55% catechins, and 45% epigallocatechin-3-gallate (EGCG), which was dissolved in 10 ml of double-distilled water and was mixed thoroughly overnight at 25–30 °C with vigorous shaking in rotary shaker. The solution was then filtered using Whatman filter paper (number 1). The GT doses of low and high concentration for GT-treated groups were then prepared using the filtrate according to body weight of the animals [12].

### Animals and treatment

**Fig. 1:**
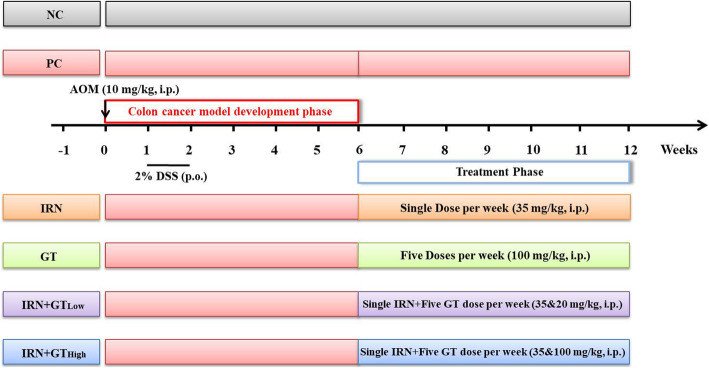
Experimental design describing the groups, dose, route of administration, and treatment period

Male Swiss albino mice (*Mus musculus* L.) were used during present study. The animals (*n* = 30) weighing approximately 6 weeks of age were procured from stock animal facility of the institute and randomly divided into six groups (negative control, positive control, IRN, GT, IRN+GT_Low_, and IRN+GT_High_) containing five mice per group. The animals were acclimatized to the laboratory condition prior to treatment and given food and water ad libitum throughout the experiment period. The test substance was dissolved in double-distilled water and applied through intraperitoneal injections to each animal, except the negative control group animals. The treatment was continued for 12 weeks in total which includes 6 weeks of colon cancer induction period and 6 weeks of treatment period. The experiment was designed in such a way that IRN was administered once a week at a dose of 35 mg/kg in both IRN and IRN+GT groups. On the other hand, GT was administered at a dose of 20 mg/kg in the IRN+GT_Low_ group and 100 mg/kg in GT and IRN+GT_High_ group for 5 days in a week consecutively for 6 weeks. After 12 weeks of continuous treatment, animals from all the groups were sacrificed by exsanguinations under Ketamine hydrochloride anesthesia (Fig. 1). Collection of blood was done in Ethylenediaminetetraacetic acid (EDTA) tubes for hematology, ALT, and serum creatinine activity. The body weights of all animals at the start of the experimentation and at the time of termination of experiment (week 12) were recorded weekly [13].

*Group I*. Distilled water

*Group II*. Azoxymethane (AOM), 10 mg/kg, 2% dextran sulfate sodium (DSS)

*Group IIA*. Distilled water

*Group IIB*. Irinotecan 35 mg/kg

*Group IIC*. Green tea 100 mg/kg

*Group IID*. Irinotecan 35 mg/kg + green tea 20 mg/kg

*Group IIE*. Irinotecan 35 mg/kg + green tea 100 mg/kg

### Gross macroscopic morphological observations of colon

Due to the treatment of AOM and DSS in positive control group, tumors are developed in both proximal and distal regions of the colon which can be observed macroscopically. The number of tumors noted from the positive control group and other treated groups and analyzed statistically for the evaluation of treatment efficacy. The length of entire colon is measured and compared with other treated groups for the difference.

### Survival

Survival of the individual after treatment of a particular disease signifies effectiveness of the treatment. The more survival percentage, the more effective is the result. Survival percentage of the treatment is analyzed using Kaplan-Meier survival curve in Graphpad Prism 5.0 software.

### Hematology

Blood collected in EDTA tubes was used for total count of WBC, neutrophil, serum ALT, and serum creatinine activity. All the hematology procedure was done manually and completed within the same day of blood collection. Total count of WBC and neutrophil was done according to Dacie and Lewis [14]. Counting of WBCs was done in Neubauer hemocytometer subjected to 2 slides per animal per group.

### Alanine transaminase (ALT) and creatinine activity

Level of serum ALT was determined using ALT kit by colorimetric method [15]. Samples of serum were incubated with either L-alanine or *α* ketoglutarate for ALT determination. The pyruvate so formed was then reacted with 2,4-dinitrophenyl hydrazine to form an adduct which absorbs light at 505 nm.

Estimation of creatinine level was performed by alkaline picrate colorimetric method in accordance with creatinine kit where values were expressed as mg% [16]. Picric acid in alkaline medium reacts with creatinine to form an orange colored complex with the alkaline picrate. Intensity of the color formed is directly proportional to the amount of creatinine present in the sample which absorbs maximum light at 520 nm.

### Histopathological studies

For histopathological studies, tissues were collected from the colon, liver, and kidney of both control and test animals. The tissues were cut into pieces of adequate size and fixed in 10% neutral-buffered formalin. The tissues were then rehydrated, washed thoroughly in tap water, dehydrated, cleared in xylene, and embedded in melted paraffin following the routine procedure. Sections were cut at 5-μm thickness and stained by routine hematoxylin and eosin (H&E) method.

### Measure of tumor volume

Measure of tumor volume in positive control group and other treated groups are important to study the efficacy of GT catechins in combination with IRN in colon cancer model. The size and volume of the tumors are measured using image J software in Leica Microsystems, Germany (DM 2000B).

### Statistical analysis

All data were presented as mean ± SEM. Statistical analyses were performed using one-way analysis of variance (ANOVA). A *p*-value < 0.05 were taken into consideration for determining significance. All statistical procedures were computed using Graphpad Prism 5.0 software.

## Results

### Body weight

**Fig. 2:**
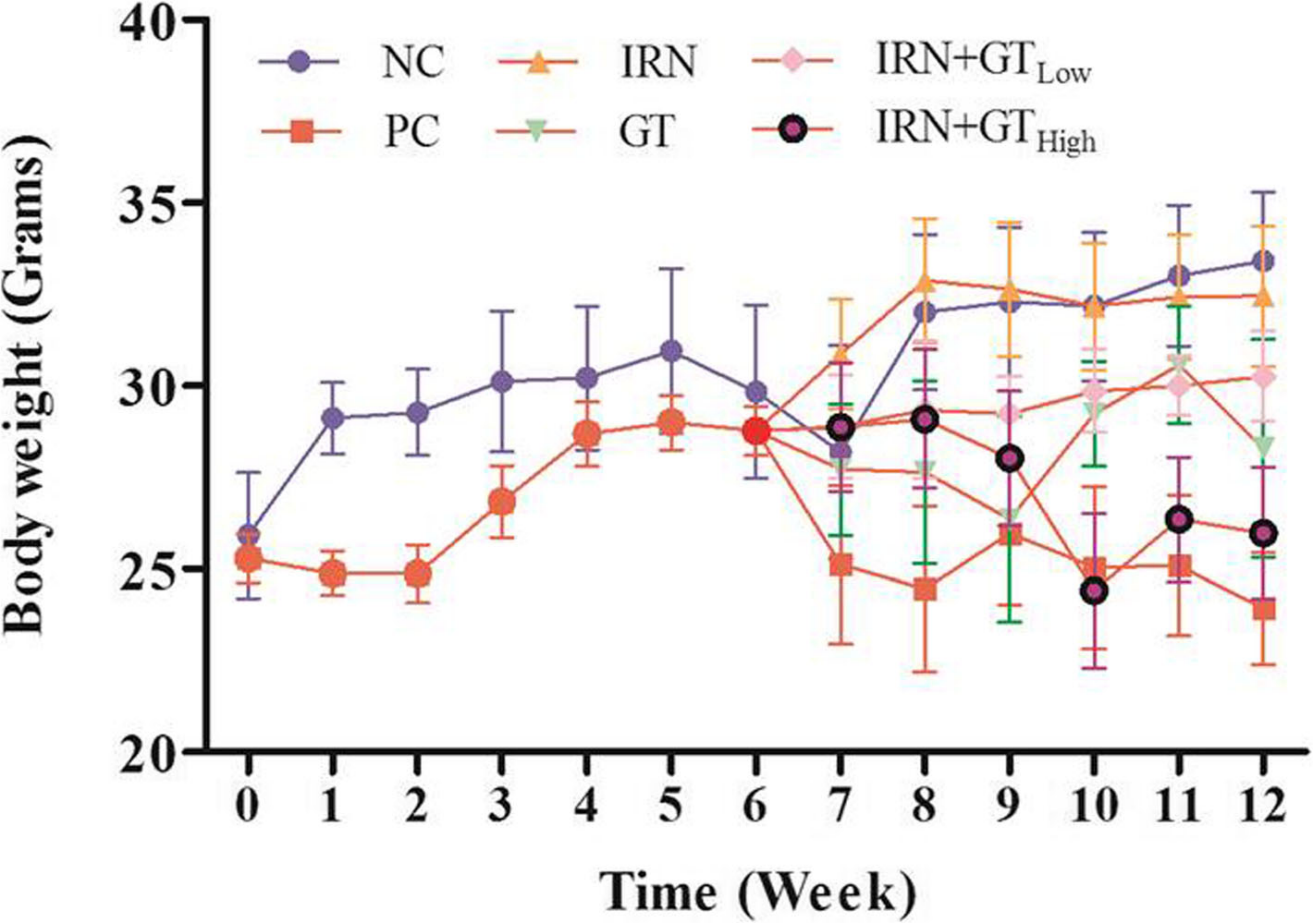
Effect of IRN and GT on body weight gain during the 12-week treatment period. Values were presented as mean ± SEM, ^#^*p* < 0.05, ^##^*p* < 0.01, and ^###^*p* < 0.01 when compared to the PC group and *p < 0.05, **p < 0.01, and ***p < 0.001 when compared to the IRN group. Increase in bodyweight was recorded in the IRN group when compared to the PC group after 12 weeks. Body weight gain was recorded to be minimal in the IRN + GT_High_ group

The positive control (PC) group expressed significant reduction in body weight as compared to negative control (NC) group in the 12-week study period, while, in comparison to the PC group, the body weight was seen to increase significantly in the IRN-treated group. Moreover, when compared to the IRN-treated group, the combined treated group showed reduced bodyweight, whereas no changes in bodyweight were observed in GT group (Fig. 2).

### Diarrhea score

The highest frequency of diarrhea was observed in the IRN-treated group when compared to the NC and PC groups. The frequency of diarrhea in combined treated groups was lower as compared to the IRN-treated group. However, the difference observed was not significant.

### Survival percentage

**Fig. 3:**
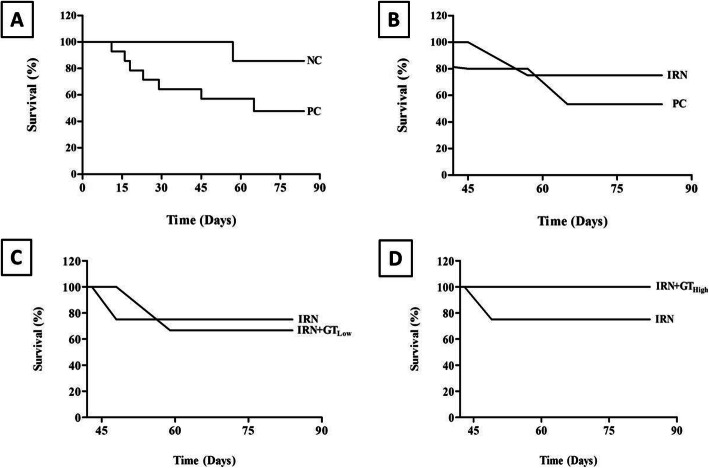
Effect of IRN and GT on survival percentage during the 12-week treatment period. Values were presented as mean ± SEM, ^#^*p* < 0.05, ^##^*p* < 0.01, and ^###^*p* < 0.01 when compared to the PC group and *p < 0.05, **p < 0.01, and ***p < 0.001 when compared to the IRN group. Significant increase in survival percentage was recorded in the IRN group when compared to the PC group. Survival percentage was recorded to be higher in the IRN+GT_High_ group

During the entire experimental period, significant reduction in survival percentage was recorded in the PC group as compared to the NC group. While increased percentage of survival was seen in the IRN-treated group when compared to the PC group. In GT and IRN+GT groups, no difference in survival percentage was marked as compared to the IRN-treated group (Fig. 3).

### Tumor number and tumor volume

**Fig. 4:**
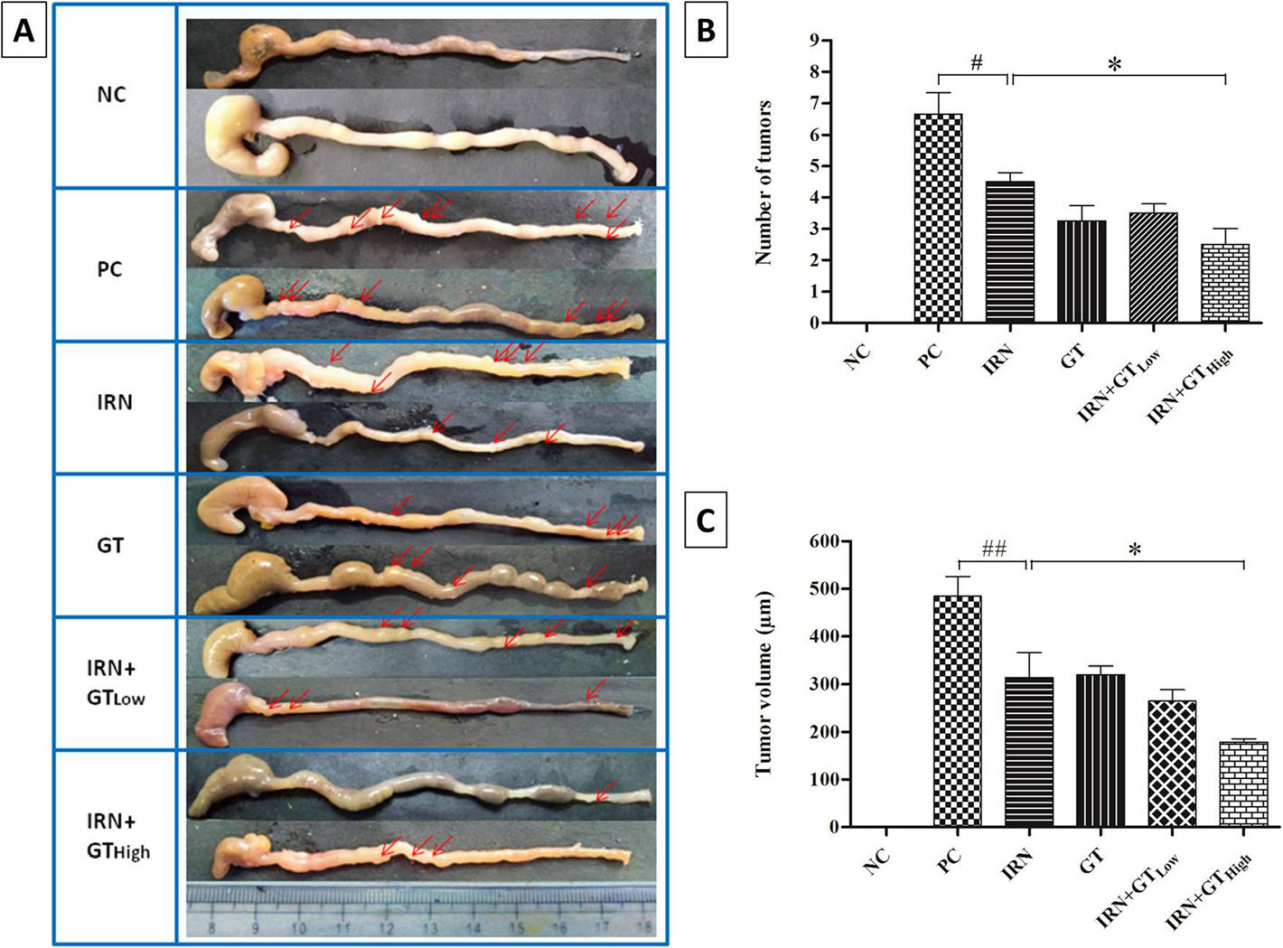
Effect of IRN and GT on **A**, **B** tumor number and tumor volume (**C**) during the 12-week treatment period. Values were presented as mean ± SEM, ^#^*p* < 0.05, ^##^*p* < 0.01, and ^###^*p* < 0.01 when compared to the PC group and *p < 0.05, **p < 0.01, and ***p < 0.001 when compared to the IRN group. Tumor number was recorded to be less in the IRN+GT_High_ group as compared to the IRN group. Significant decrease in tumor volume was recorded in the IRN+GT_High_ group as compared to the IRN group

The highest number of tumors was recorded in the PC group. When IRN-treated group was compared to the PC group, significant reduction in tumor numbers was observed. In the IRN+GT_High_ group, the number of tumors was seen to decrease significantly as compared to the IRN-treated group (Fig. 4). The tumor volume in the IRN+GT_High_ group was found to be significantly reduced when compared to the IRN group (Fig. 4).

### Hematology

**Fig. 5:**
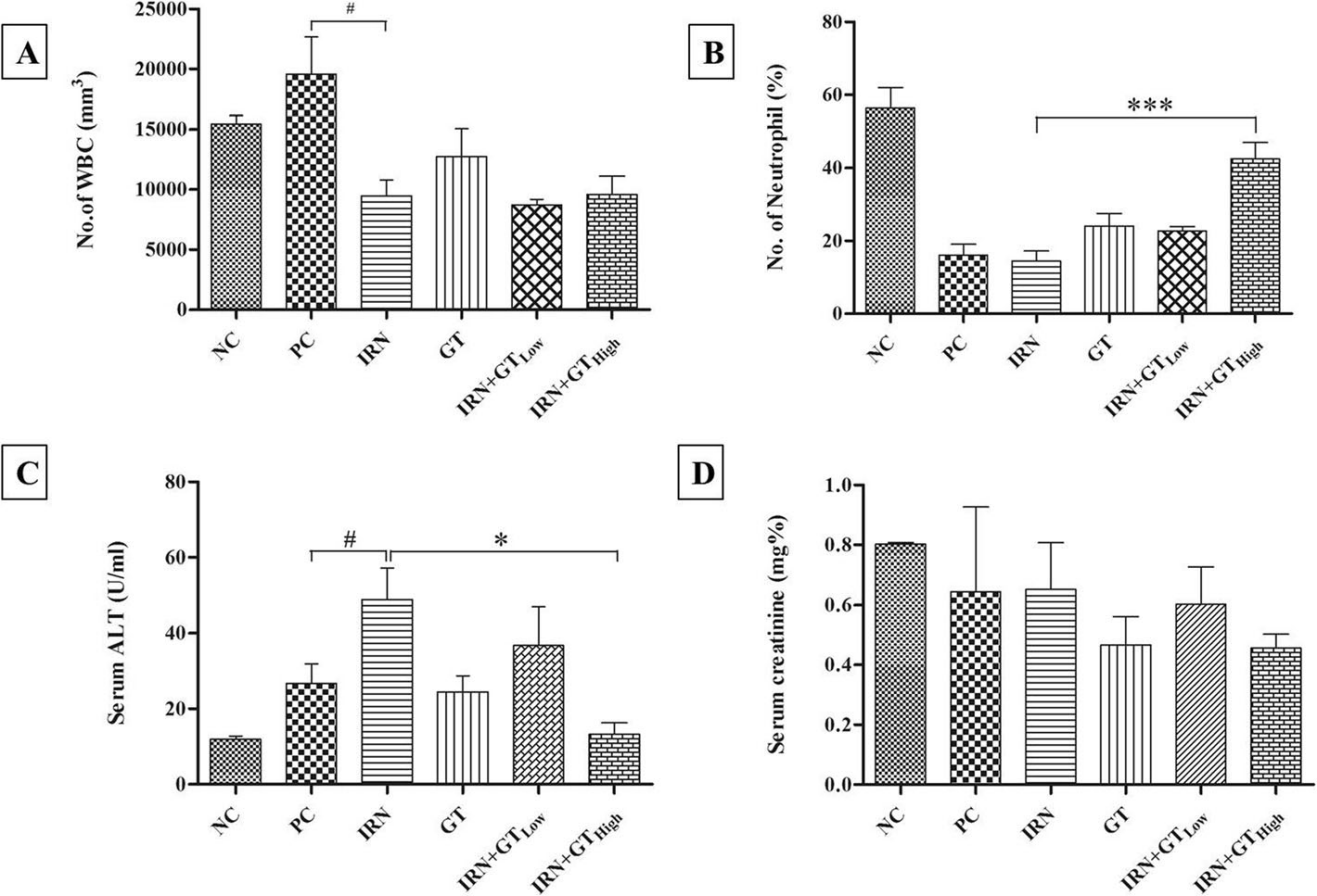
Effect of IRN and GT on **A** total number of WBC, **B** percentage of neutrophil, **C** serum ALT, and **D** serum creatinine level during the 12-week treatment period. Values were presented as mean ± SEM, ^#^*p* < 0.05, ^##^*p* < 0.01, and ^###^*p* < 0.01 when compared to the PC group and *p < 0.05, **p < 0.01, and ***p < 0.001 when compared to the IRN group. Decrease in number of WBC was recorded in treated groups; however, decrease was recorded to be significant and maximum in the IRN group as compared to the PC group. Significant increase in number of neutrophil was recorded in the IRN+GT_High_ group as compared to the IRN group. Significant increase in serum ALT level was recorded in the IRN group when compared to the PC group. ALT level was recorded to be less in the IRN+GT_High_ group as compared to the IRN group. Decrease in serum creatinine level was recorded in treated groups; however, decrease was recorded to be maximum in the IRN+GT_High_ group

Increase in WBC count was observed in the PC group as compared to the NC group. When compared to the PC group, significant reduction in the number of WBC was seen in the IRN-treated group. An increase in total number of WBC was recorded in the IRN+GT_High_ group as compared to the IRN group. However, the difference was not significant (Fig. 5a).

As compared to the NC group, the number of neutrophil was found to be reduced significantly in the PC and IRN group. While, the total number of neutrophil significantly increased in the IRN+GT_High_ group when compared to the IRN group (Fig. 5b).

### Serum ALT and creatinine Level

In this study, serum ALT level did not show significance difference in the PC group when compared to the NC group. However, when IRN group was compared to the PC group, significant increase in ALT level was observed. The level of serum ALT showed significant reduction in the IRN+GT_High_ group as compared to the IRN group (Fig. 5a).

In the serum creatinine level, as compared to the PC group, no significant difference was noted in the treated groups, while the serum creatinine level was found to be higher in the IRN group in contrast to the IRN+GT_High_ group with decreased level of creatinine (Fig. 5b).

### Histopathology

**Fig. 6:**
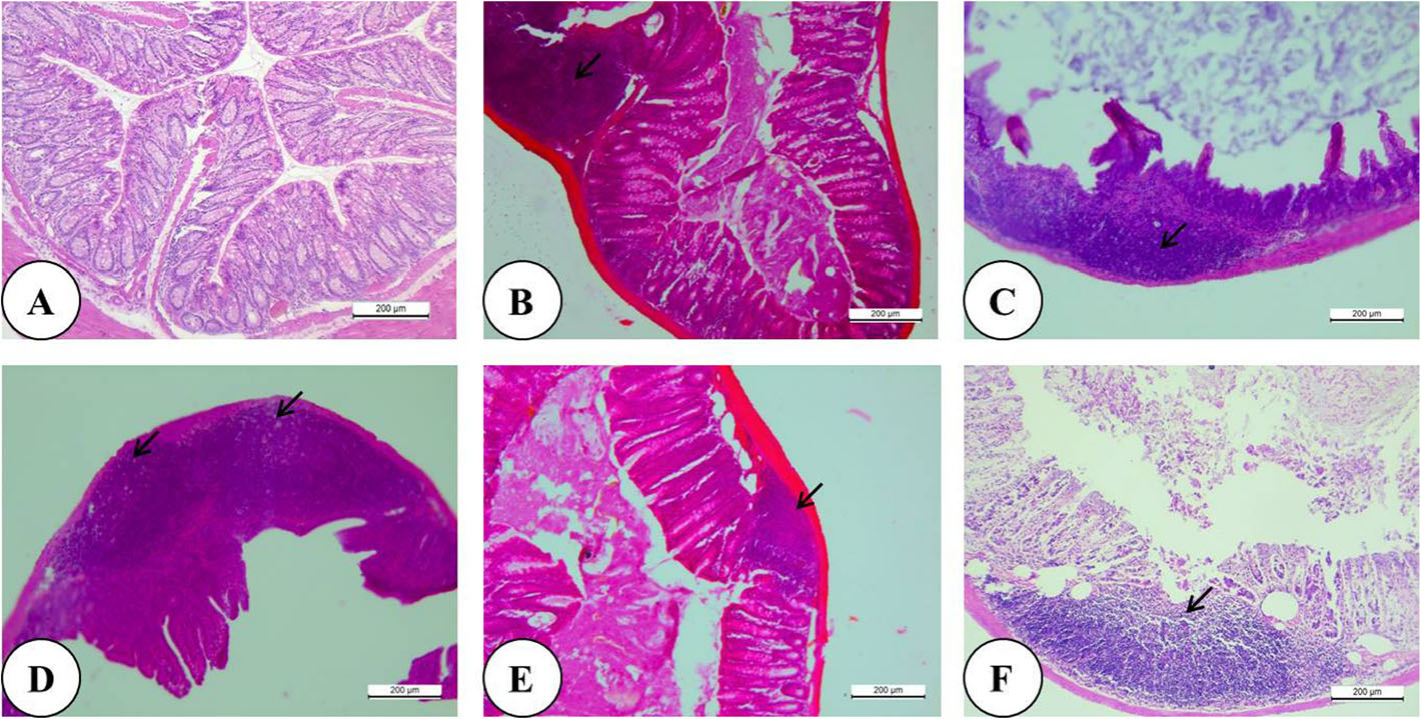
Histopathology of colon tissue sections stained with hematoxylin and eosin. **A** NC shows normal colonic mucosa. **B** PC group with maximum tumoral growth in the 12-week study period. **C** IRN group showing reduction in tumoral growth. **D** GT group showing higher tumor growth as compared to the IRN group. **E** IRN+GT_Low_ group showing decrease in tumor growth as compared to the IRN group. **F** IRN+GT_High_ group showing decrease in tumor growth as compared to the IRN group along with cellular apoptosis. (arrow head represents area with tumor growth without adenomatous differentiation)

**Fig. 7:**
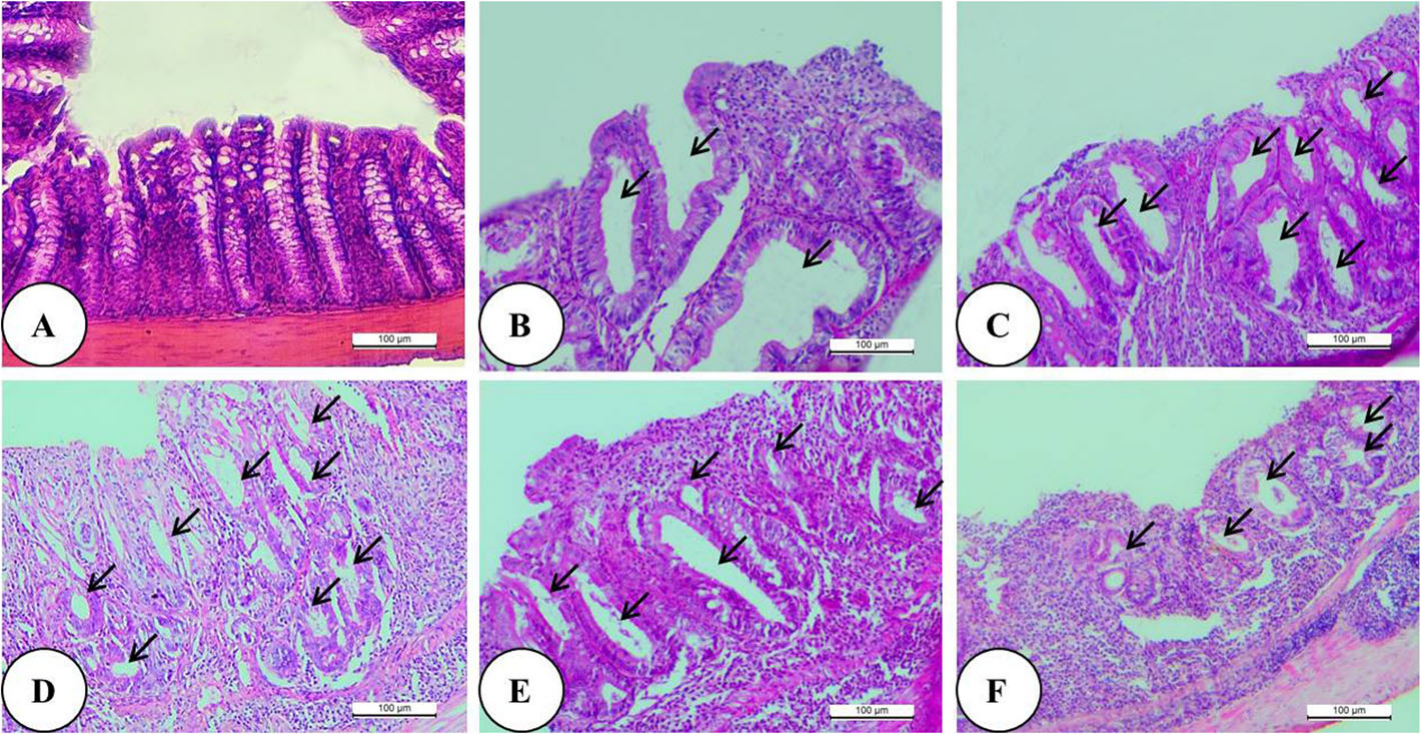
Histopathology of colon tissue sections stained with hematoxylin and eosin. **A** NC shows normal colonic mucosa. **B** PC group with maximum tumoral growth with tubular adenocarcinoma in the 12-week study period. **C** IRN group showing reduction in adenocarcinomatous growth. **D** GT group showing higher number of tubular adenocarcinoma as compared to the IRN group. **E** IRN+GT_Low_ group showing decrease in tubular adenocarcinoma as compared to the IRN group. **F** IRN+GT_High_ group showing decrease in tubular adenocarcinoma as compared to the IRN group along with cellular apoptosis. (Arrow head represents differentiation of tubular adenocarcinoma)

In the PC group, severe inflammatory lesions was observed in the form of crypt loss, accompanied by surface erosion with exuberant inflammatory exudates, patchy re-epithelization, lamina propia fibrosis with acute and chronic inflammatory infiltrate, submucosal edema, and mixed inflammatory cell infiltrate. Additionally, developed adenomatous characters including differentiation in the form of tubular adenocarcinoma were noted. The lesions were well demarcated from normal mucosa with the cells displaying atypia with considerable variation in size of both cells and nuclei. The lesions were expansile and compressed the surrounding tissue. Lesions clearly invading the muscularis mucosa into the submucosa were classified as carcinomas, while those without infiltrative growth were classified as adenomas. Decrease in inflammatory cell infiltrate along with cellular apoptosis was observed in the IRN+GT_High_ group as compared to the IRN group (Fig. 6). The IRN group showed decreased tubular adenocarcinoma as compared to the PC group. In the IRN+GT_High_ group, reduced differentiation in tubular adenocarcinoma was observed with decreased inflammatory cell infiltration as compared to the IRN group (Fig. 7).

**Fig. 8:**
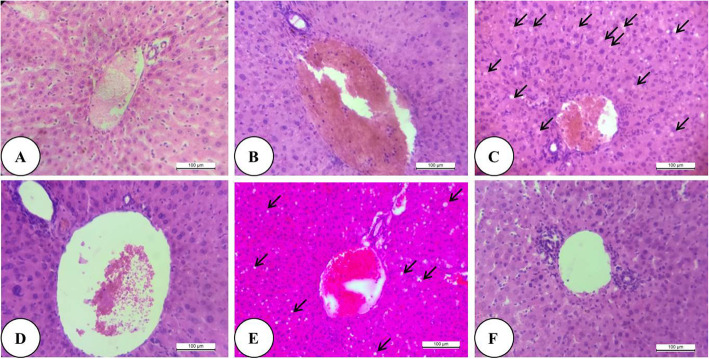
Histopathology of liver tissue sections stained with hematoxylin and eosin. **A** NC group and **B** PC group shows normal liver histology with central vein and hepatocytes. **C** IRN group low-grade NAFLD in the form of fat accumulation in the liver tissue in the 12-week study period. **D** GT group showing normal liver histology. **E** IRN+GT_Low_ group showing decrease in fat accumulation as compared to the IRN group. **F** IRN+GT_High_ group showing normal liver histopathology without NAFLD. (Arrow head represents area with fat accumulation)

Histopathology of liver in the NC group exposed normal hepatocytes along with connective tissue, stroma, blood vessels, nerves, lymphatic vessel, and bile duct. As compared to the NC group, similar morphology was seen in the PC group. But, in the IRN group, moderate hepatic steatosis was observed in contrast to the PC group. In the IRN+GT_High_ group, no hepatic steatosis was marked following normal histomorphology of liver (Fig. 8).

**Fig. 9:**
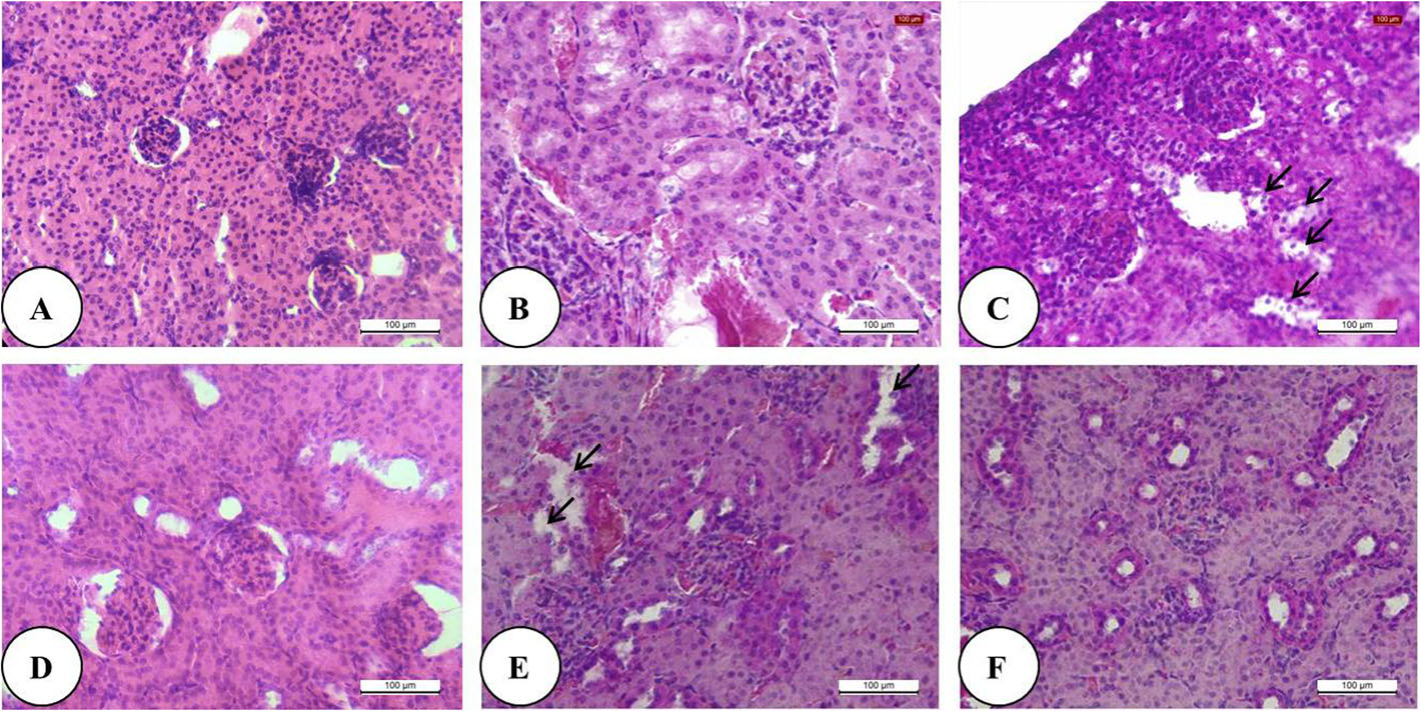
Histopathology of kidney sections stained with hematoxylin and eosin. **A** NC group and **B** PC group showing normal kidney histology. **C** IRN group showing mild tubular damage. **D** GT group showing normal kidney histology. **E** IRN+GT_Low_ group showing decrease tubular damage as compared to the IRN group. **F** IRN+GT_High_ group showing normal kidney histology. (Arrow head represents tubular damage)

The kidney tissue stained with hematoxylin and eosin evinced distinct architecture displaying kidney tubules, glomerulus, and Bowman’s capsule. Damages in these parts were considered for determination of injury. In this study, mild tubular damage was observed in the IRN group as compared to the NC and PC groups. However, decrease in tubular damage was observed in the IRN+GT_Low_ group as compared to the IRN group. Normal kidney histology without tubular damage was observed in the IRN+GT_High_ group as compared to the IRN group (Fig. 9).

## Discussion

Recent reports suggested close association between inflammation and cancer as malignant cells are found to be capable of local and systemic inflammation, transcription factor and inflammatory cytokine activation. Inflammatory infiltrate from peritumoral and tumoral stroma was mainly formed by lymphocytes, plasma cells, and macrophages in colon along with more number of blood capillaries especially congested capillaries [17]. Mogoantă et al. [17] also reported the higher and heterogeneous involvement of T-lymphoctes and macrophages in the tumors of colon carcinoma and lesser involvement of B-lymphocytes. According to Tanaka et al. [18] treatment with AOM and DSS can induce inflammation-associated colon adenocarcinoma in 4 weeks which mimics colon cancer and can serve as model for experimental studies. In the present study, higher inflammatory infiltration was observed in 6 weeks as compared to 12 weeks with higher differentiation of adenocarcinoma in contrast to the the 6-week study period. The intensity of inflammatory response was found to be different from one group to another. The number of tumors in the PC group was found to be the highest as compared to other groups in the 12-week study period.

IRN is a potent inhibitor of topoisomerase-I which reduces the number of tumors in the IRN group with moderate inflammatory infiltration associated with adenomatous differentiation and cellular apoptosis. The cytotoxic mechanism of IRN activity was reported to be cellular apoptosis caused due to prevention of relegation in single strand break during S phase of cell cycle [19]. On the other hand, various studies corroborated the potency of GT in the treatment of colon cancer and evinced the involvement of cellular apoptosis by GT in the disease pathology [20]. Besides, Kondo et al. [13] reported the role of antioxidant in colon cancer therapy in combination with IRN and demonstrated the significance of antioxidants in therapeutic interventions. In our study, it was found that IRN significantly reduced the number of tumors in treated animals when compared to the PC group and decreased adenomatous differentiation was recorded in the IRN-treated group. However, it was also discovered that addition of GT 100 mg/kg along with IRN (IRN+GT_High_ group) increases the treatment efficacy significantly in terms of tumor number, adenomatous differentiation and tumor volume. The number of apoptotic cells in the tumor area was also recorded to be higher in this group. This difference was not observed when 20 mg/kg of GT was injected along with IRN (IRN+GT_Low_ group).

However, experimental evidences highlighted severe toxicities associated with IRN in cancer therapy which includes leucopenia as one of the common side effects of IRN treatment [21]. Paulik et al. [6] reported the risk of neutropenia from IRN treatment by reducing the number of neutrophil and leucocyte in the peripheral blood which is a marked feature of IRN toxicity. However, Elkirdasy et al. [22] examined the effect of GT extracts and demonstrated increased percentage of neutrophil in WBC to the control level in experimental animals. In this study, administration of IRN reduced the number of leucocytes as well as neutrophils which is a marked effect of IRN toxicity. It was recorded that, combination of GT catechins along with IRN treatment in the IRN+GT_High_ group increases the number of both leucocyte and neutrophil. However, significant difference was observed in case of increased neutrophil count. Nonetheless, neutrophils have evident anticancer activity by serving as anticancer immunity and have been reported to initiate phagocytosis as well as trogocytosis accompanied by cytotoxic obliteration of cancer cells [23]. Increasing the number of neutrophil, GT plays significant role in reducing the toxicity of IRN and increases efficacy of the cancer treatment.

Chemotherapy-based liver injury in colorectal cancer includes NAFLD when IRN-based regimens are applied for the treatment [24]. Steatosis is the aggregation of lipids within the hepatocytes and is considered the preliminary stage of NAFLD [25]. Both in vivo and in vitro studies suggested that GT catechins are likely to prevent steatosis by decreasing intestinal lipid and carbohydrate absorption along with adipose lipolysis, hepatic B-oxidation, thermogenesis, and insulin sensitivity [26]. In this study, hepatic steatosis was recorded in the IRN-treated group as a dose-limiting side effect of IRN. This effect of IRN was found to be ameliorated in the IRN+GT_High_ group when observed histopathologically. Elevation in serum ALT is a marked feature of liver damage and higher level of ALT was found in the IRN-treated group which was further reduced due to GT administration. In case of serum creatinine level, creatinine clearance was observed maximum in the IRN+GT_High_ group; however, no significant difference was observed among the treated groups as such.

Body weight loss is reported to be another marked feature of colon carcinogenesis [27]. In the PC group, significant decrease in body weight was recorded as compared to the NC group. However, in the IRN group, the body weight was seen to increase as compared to the PC group. Since GT was reported for its ability to enhance energy expenditure, fat oxidation, and reduction in body weight, [28] increase in body weight was minimal in GT-administered combined treated groups. In terms of kidney functions, IRN was reported to cause dose-limiting nephrotoxicity during treatment [29]. On the other hand, GT was found to reduce blood urea nitrogen, creatinine levels, and improve renal function in different disease condition [30]. In the present study, it was observed that treatment with IRN induces tubular damage and addition of GT along with IRN ameliorates such damages when analyzed histologically. However, significant difference was not established when serum creatinine level was measured.

## Conclusion

Based on our findings, it can be concluded that combined treatment of IRN and GT improves the disease conditions in colon cancer mouse model. Along with antitumor effects, GT also ameliorates treatment-associated side effects of IRN. Hence, it is beneficial to introduce GT as treatment regimen with IRN for ameliorative potency and lower side effects. The efficacy of GT is found to be conducive in IRN therapy and the aspects of combination in drug formulation of both GT and IRN could open up a new era of therapeutic insight for more accurate and conclusive therapy for colon cancer.

## Data Availability

The raw data and materials are available upon request from corresponding author.

## Abbreviations

IRN: Irinotecan
GT: Green tea
NAFLD: Non-alcoholic fatty liver disease
AOM: Azoxymethane
DSS: Dextran sulfate sodium
EGCG: Epigallocatechin-3-gallate
WBC: White blood cells
ALT: Alanine transaminase
EDTA: Ethylenediaminetetraacetic acid
NC: Negative control
PC: Positive control
